# Impact of hemodialysis on the concentrations of sodium and potassium during infusion of sodium thiosulfate using an *In Vitro* hemodialysis model

**DOI:** 10.1371/journal.pone.0224767

**Published:** 2019-11-13

**Authors:** Sagar U. Nigwekar, Amy Barton Pai, Bruce Mueller, Michael C. Dean, Gabrielle Costello, Craig R. Sherman

**Affiliations:** 1 Division of Nephrology, Department of Medicine, Massachusetts General Hospital, Boston, MA, United States of America; 2 University of Michigan College of Pharmacy, Ann Arbor, MI, United States of America; 3 Hope Pharmaceuticals, Scottsdale, AZ, United States of America; University of Mississippi Medical Center, UNITED STATES

## Abstract

**Introduction:**

The purpose of this study was to evaluate the impact of hemodialysis on the concentrations of sodium and potassium in the blood when a 25 g dose of sodium thiosulfate injection is infused over 60 minutes in combination with hemodialysis.

**Methods:**

Sodium thiosulfate (25 g) was prepared by diluting 100 mL of 250 mg/mL Sodium Thiosulfate Injection with 800 mL of 5% dextrose. This was added to the circulating blood surrogate solution at a rate of 15 mL/minute using an infusion pump of an *in vitro* model of dialysis machine. Serial samples were collected before the administration of the sodium thiosulfate solution, after 15 minutes, 30 minutes, and 60 minutes of infusion from pre-and post-dialyzer ports in both the dialysate circuit and the extracorporeal circuit.

**Findings:**

The concentration of sodium thiosulfate in pre-dialyzer and post-dialyzer samples of the circulating blood surrogate solution peaked at 30 minutes and 15 minutes, respectively and then remained relatively unchanged during the remainder of the infusion. Mean sodium concentrations (mEq/L) in the circulating blood surrogate solution collected after exposure to a dialyzer were 103.2 ± 12.2, 114.2 ± 18.8, 117.2 ± 7.5, 93.5 ± 5.9 at 0, 15, 30, and 60 minutes, respectively (p = 0.248). Mean potassium concentrations (mEq/L) in the circulating blood surrogate solution collected after exposure to a dialyzer were 1.4 ± 0.3, 1.6 ± 0.3, 1.5 ± 0.1, 1.2 ± 0.1 at 0, 15, 30, and 60 minutes, respectively (p = 0.365). Sodium and potassium concentrations in dialysate increased marginally after exposure to the dialyzer.

**Discussion:**

Our study demonstrates that neither potassium nor sodium accumulated in circulating blood surrogate solution when a dose of sodium thiosulfate was infused in conjunction with hemodialysis.

## Introduction

The United States Food and Drug Administration (FDA) has approved Sodium Thiosulfate Injection for the treatment of acute cyanide poisoning that is judged to be serious or life-threatening. In patients with cyanide poisoning, sodium thiosulfate serves as a sulfur donor for rhodanese, an enzyme that detoxifies cyanide by transforming it to thiocyanate.[1] Thiocyanate is subsequently excreted in urine. Adverse effects from sodium thiosulfate injection when administered as a treatment for cyanide poisoning are rare.[2]

A number of retrospective studies and case reports suggest intravenous sodium thiosulfate may be a potentially effective treatment for calciphylaxis, a rare but serious vascular calcification disorder predominantly seen in patients with dialysis-dependent end-stage kidney disease.[3–8] Possible mechanisms of action of sodium thiosulfate in calciphylaxis include vasodilation, direct calcification inhibition, and anti-oxidant actions.[3, 4] Sodium Thiosulfate Injection is currently being evaluated in a phase 3 clinical study for the treatment of calciphylaxis in patients with end stage kidney disease who receive chronic hemodialysis (Clinicaltrials.gov ID: NCT03150420).[9] Participants randomized to the active intervention arm in this trial receive intravenous infusion of 100 mL of 250 mg/mL (25 g) Sodium Thiosulfate Injection (Hope Pharmaceuticals) mixed with 800 mL of 5% dextrose solution during the last 60 minutes of hemodialysis session three times a week for three weeks. The FDA-approved Sodium Thiosulfate Injection contains 250 mg of sodium thiosulfate per mL. Sodium represents approximately 29% of the molecular weight of anhydrous sodium thiosulfate; therefore, each mL of the medication contains 72.5 mg of sodium. Each mL of the drug product also contains 4.4 mg of potassium chloride.^10^ Potassium represents approximately 52% of the molecular weight of potassium chloride; therefore, each mL of the medication contains 2.3 mg of potassium.[10] A 25 g dose of the medication (100 mL) contains approximately 7.25 g of sodium and 230 mg of potassium. This amount of potassium is comparable to the potassium content that may be present in one unit of packed red blood cells.[11, 12] Additionally, this amount of potassium is a fraction of the 1560–4680 mg of potassium that can be removed by hemodialysis during a typical dialysis session that lasts between 3 and 5 hours.[13]

Sodium and potassium accumulation leading to volume overload and hyperkalemia, respectively are major adverse consequences of renal failure. The purpose of this study was to evaluate the impact of hemodialysis on the concentrations of sodium and potassium in the blood when a 25 g dose of Sodium Thiosulfate Injection is infused over 60 minutes in combination with hemodialysis using an *in vitro* hemodialysis model. We hypothesized that there will be no significant accumulation of sodium or potassium in circulating blood surrogate solution when sodium thiosulfate is infused in conjunction with hemodialysis as hemodialysis will remove the incremental exposures to sodium and potassium associated with sodium thiosulfate therapy.

## Materials and methods

The procedures in this study did not involve any human or animal experiments. Ethics approval, therefore, was not necessary.

### *In vitro* model

**Fig 1:**
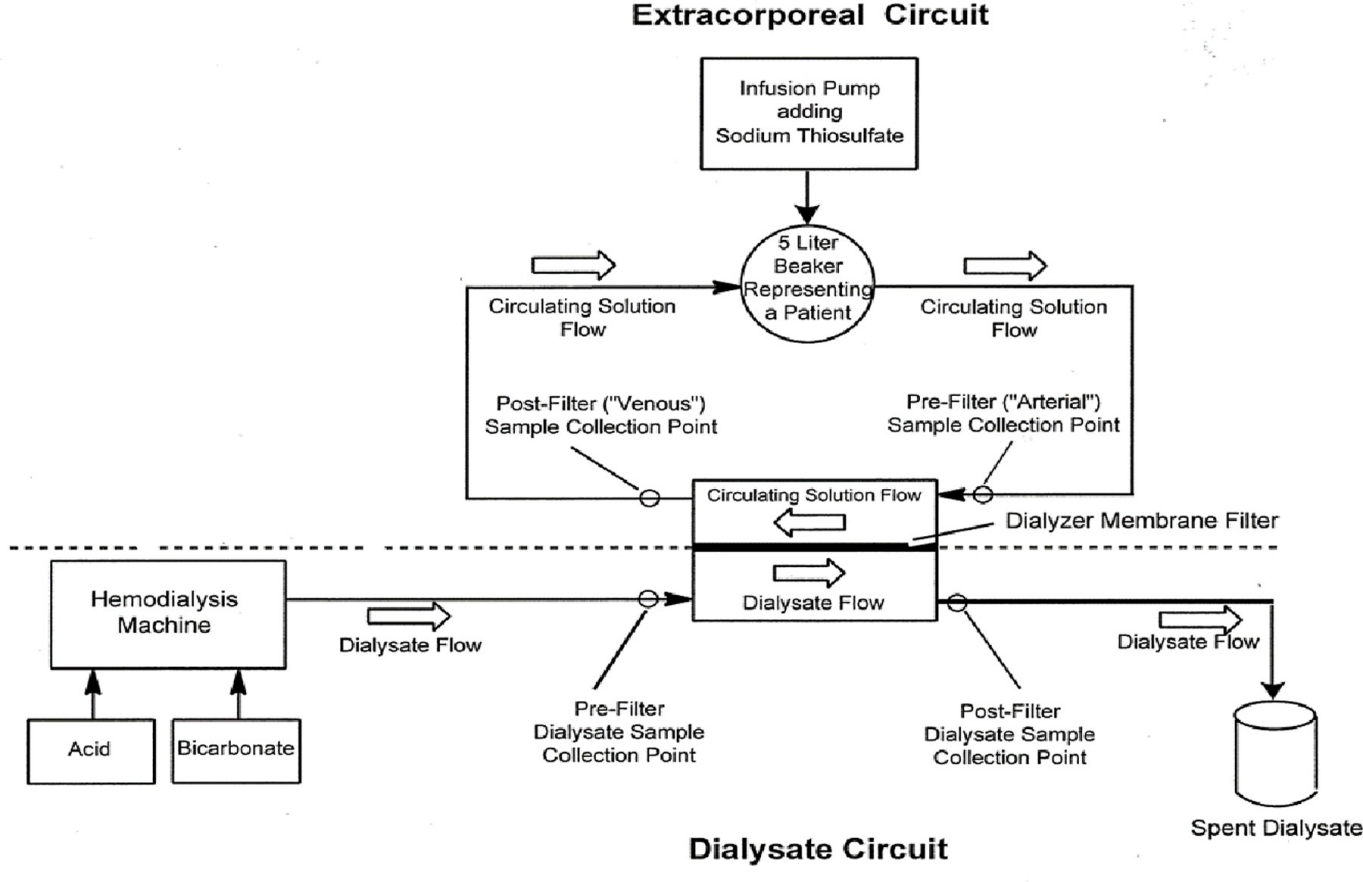
Schematic diagram of *in vitro* hemodialysis model.

The *in vitro* model utilized a commercially available Fresenius 2008T dialysis machine (Fresenius Medical Care, Waltham, MA) connected to a reverse osmosis system for water purification (Fig 1). The dialysate circuit was connected using a compatible Fresenius hemodialysis blood tubing set primed with dialysate and a high permeability (high flux) polysulfone hemodialyzer (Fresenius Optiflux^®^ F200; K_uf_ = 74 mL/h/mmHg; surface area = 1.9 m^2^). Dialysate with target concentrations of 137 mEq/L sodium and 2 mEq/L potassium was prepared using manufacturer recommended Naturalyte^®^ 4000 sodium bicarbonate concentrate (Fresenius Medical Care) and Centrisol^®^ calcium-free acid concentrate Formula SB-140 (Minntech, Minneapolis, MN) diluted with deionized water in a 1:45 ratio. The dialysate flow rate was 600 mL/minute. Details of the dialysis prescription are summarized in Table 1.

**Table 1:** Dialysis settings.

Parameter	Setting
Dialysate flow rate	600 mL/min
Ultrafiltration rate	15 mL/min
Blood flow rate	300 mL/min
Infusion pump setting	Deliver 900 mL/hour of thiosulfate solution
Infusion pump line	Connected to the blood side volume in the five litters

This model used five liters of dialysate as prepared in the dialysate circuit as a blood surrogate. The blood surrogate is continuously circulated from a 5-liter beaker, through tubing to the hemodialyzer and through additional tubing back to the 5-liter beaker. The flow rate of the circulating blood surrogate solution was 300 mL/minute.

### Sodium thiosulfate

Sodium thiosulfate (25 g) was prepared by diluting 100 mL of 250 mg/mL Sodium Thiosulfate Injection (Hope Pharmaceuticals) with 800 mL of 5% dextrose solution for a total volume of 900 mL. A typical clinical scenario of hemodialysis patients with calciphylaxis involves receiving intravenous sodium thiosulfate infused into the venous bloodline of the dialyzer during the last 30–60 minutes of a hemodialysis session.[4] To simulate this human scenario, sodium thiosulfate solution (900 ml) was added to the circulating blood surrogate solution at a rate of 15 mL/minute using an infusion pump (total infusion duration of 60 minutes). The ultrafiltration rate was set to 15 mL/minute to maintain a consistent circulating blood surrogate solution volume.

### Sample analysis

Samples (15 mL each) were collected before the administration of the sodium thiosulfate solution, after 15 minutes, 30 minutes, and 60 minutes of infusion from pre-and post-dialyzer ports in both the dialysate circuit and the extracorporeal circuit. Ultrafiltration was temporarily stopped after each sampling timepoint to restore the volume of the circulating blood surrogate solution. Samples were placed on ice and subsequently stored at -80C° until shipped for analyses.

Potassium and sodium concentrations were analyzed by ICP- Mass Spectrometry using a Perkin Elmer, NexION 300X ICP-MS instrument. Analytical samples were prepared by adding 1.0 mL of a sample solution to a 50.0 mL volumetric digitube, adding 2.0 mL of nitric acid (HNO_3_), 0.1 mL scandium (1000 ppm) as the internal standard before diluting to a final volume of 50.0 mL with purified water. Both elements were calibrated using a 4-point calibration curve. The range for the sodium curve covered 6.0 ppm to 48.2 ppm, while the potassium curve range was 0.50 ppm to 4.0 ppm.

Sodium thiosulfate concentration was analyzed by liquid chromatography. The calibration curve range included nine standards covering a range of 0.1 ppm to 50 ppm. Samples were initially analyzed as received. Some sample results were over the range of the calibration curve. Applicable samples were diluted with purified water and reanalyzed.

Concentrations of potassium, sodium, and sodium thiosulfate were measured 3 times. All values of analytes are expressed as the mean (standard deviation). Wilcoxon Scores Rank Sum test was applied to compare levels of analytes at different timepoints of sodium thiosulfate infusion. P<0.05 was considered statistically significant.

## Results

### Thiosulfate (Fig 2)

Thiosulfate was not detected in any sample that was collected before the start of the 60-minute infusion of sodium thiosulfate into the circulating blood surrogate solution. The concentration of sodium thiosulfate in pre-dialyzer (“arterial”) samples of the circulating blood surrogate solution peaked at 30 minutes with a mean value of 1035.4 μg/mL ± 189.5 μg/mL and remained elevated through 60 minutes with a mean value of 978.7 μg/mL ± 211.1 μg/mL (p = 0.016 for comparison between the baseline and mean of levels from the 15, 30 and 60 minute timepoints and p = 0.023 for comparison across the timepoints of 0, 15, 30, and 60 minutes of sodium thiosulfate infusion).

**Fig 2:**
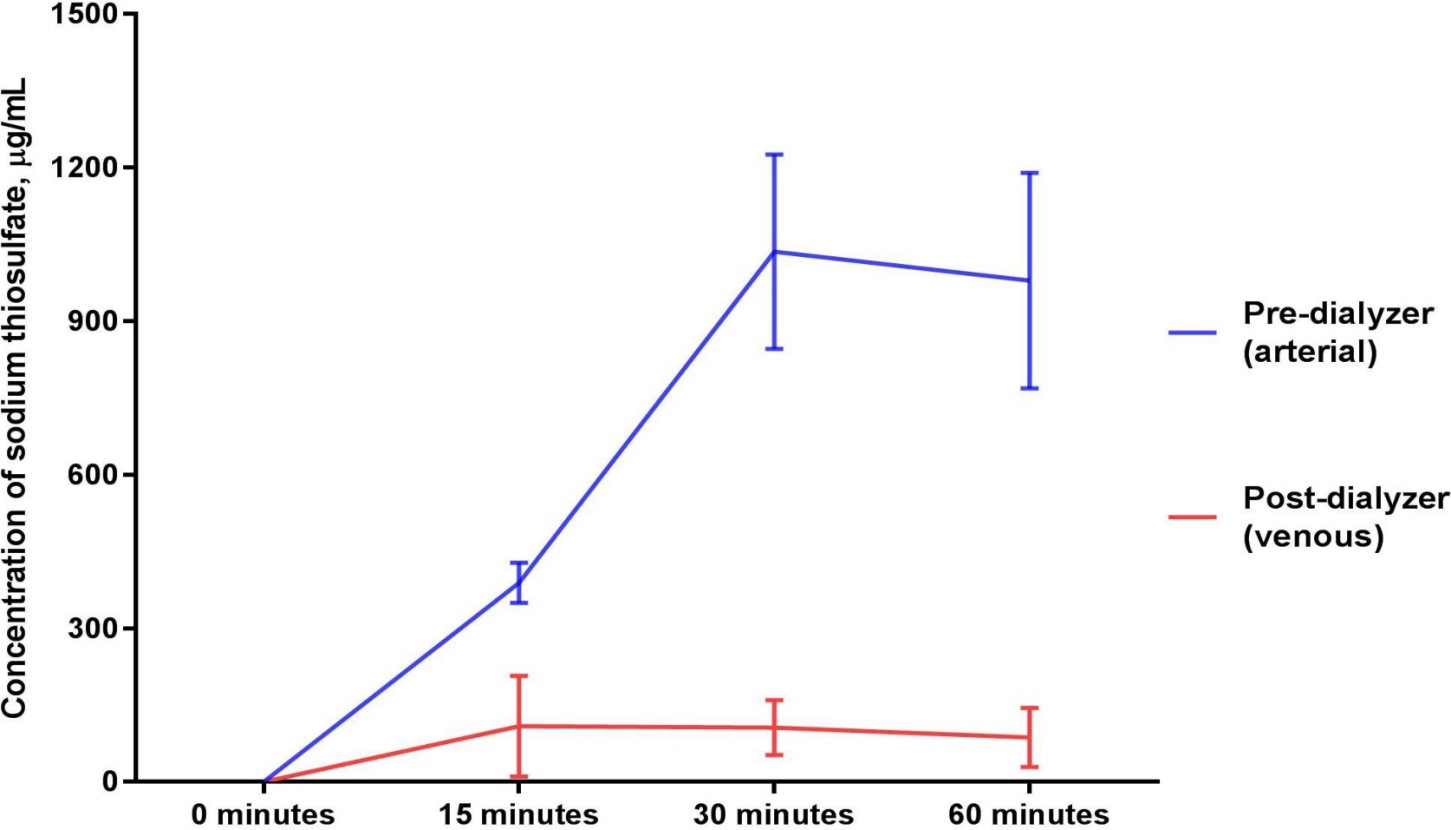
Sodium thiosulfate concentrations in the circulating blood surrogate solution before and after exposure to a dialyzer during ongoing hemodialysis and 60-minute infusion of 25 g Sodium Thiosulfate Injection.

In contrast, the concentration of sodium thiosulfate in post-dialyzer (“venous”) samples of the circulating blood surrogate solution peaked at 15 minutes with a mean value of 108.9 μg/mL ± 98.5 μg/mL and remained relatively unchanged during the remainder of the infusion over the subsequent 45 minutes (p = 0.015 for comparison between the baseline and mean of levels from the 15, 30 and 60 minute timepoints and p = 0.071 for comparison across the timepoints of 0, 15, 30, and 60 minutes of sodium thiosulfate infusion).

### Sodium (Figs 3 and 4)

Mean sodium concentrations (mEq/L) in the circulating blood surrogate solution collected after exposure to a dialyzer were 103.2 ± 12.2, 114.2 ± 18.8, 117.2 ± 7.5, 93.5 ± 5.9 at 0, 15, 30, and 60 minutes, respectively (p = 0.579 for comparison between the baseline and mean of levels from the 15, 30 and 60 minute timepoints and p = 0.248 for comparison across the timepoints of 0, 15, 30, and 60 minutes of sodium thiosulfate infusion). Mean sodium concentrations (mEq/L) in the circulating blood surrogate solution collected before exposure to a dialyzer were statistically not different (p = 0.196 for comparison between the baseline and mean of levels from the 15, 30 and 60 minute timepoints and p = 0.319 for comparison across the timepoints of 0, 15, 30, and 60 minutes of sodium thiosulfate infusion).

**Fig 3:**
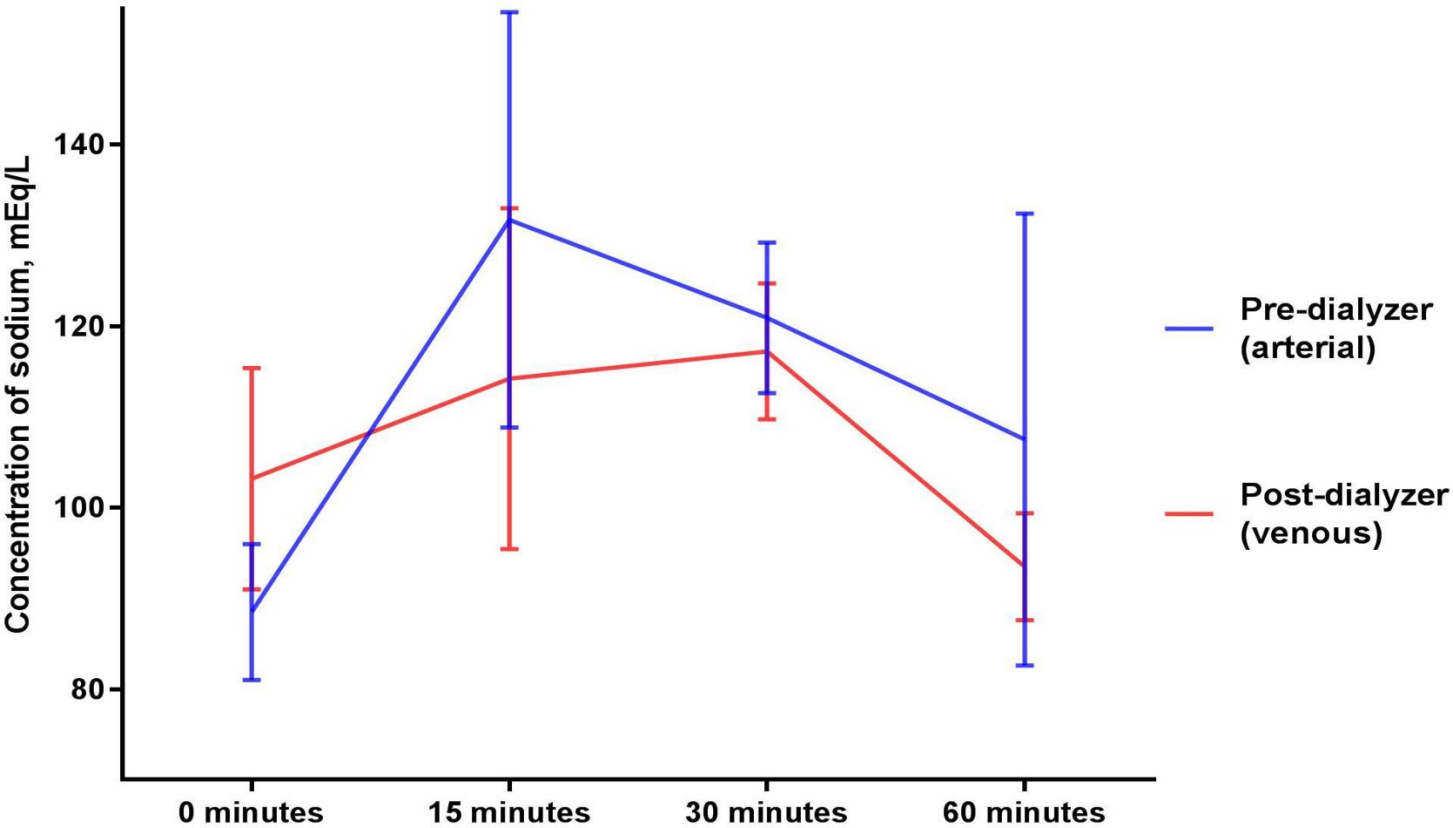
Sodium concentrations in the circulating blood surrogate solution before and after exposure to a dialyzer during ongoing hemodialysis and 60-minute infusion of 25 g Sodium Thiosulfate Injection.

**Fig 4:**
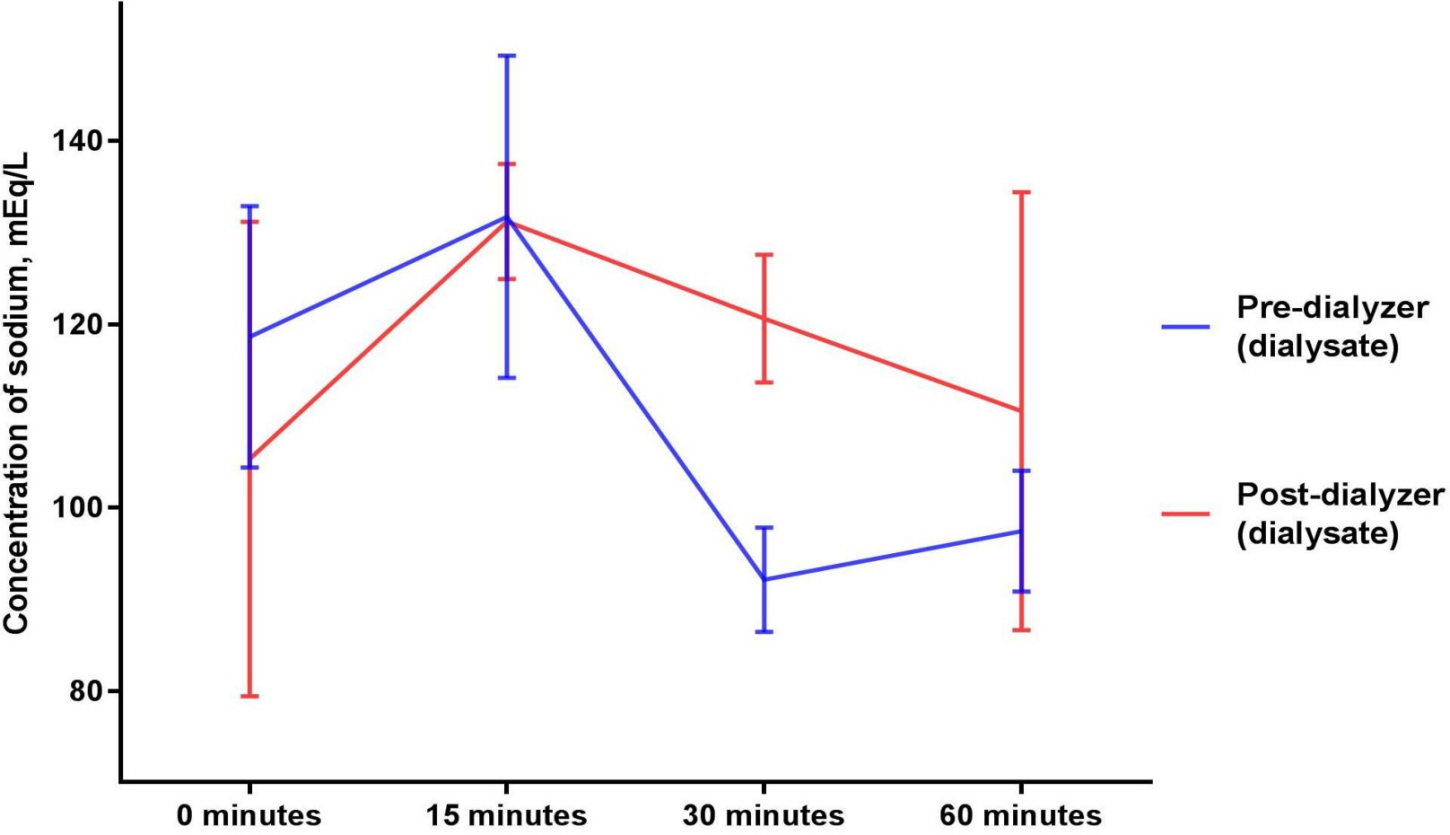
Sodium concentrations in dialysate collected before and after exposure to a dialyzer during ongoing hemodialysis and 60-minute infusion of 25 g Sodium Thiosulfate Injection.

Sodium concentrations in dialysate increased marginally after exposure to the dialyzer (p = 0.739 for comparison between the baseline and mean of levels from the 15, 30 and 60 minute timepoints and p = 0.579 for comparison across the timepoints of 0, 15, 30, and 60 minutes of sodium thiosulfate infusion).

### Potassium (Figs 5 and 6)

Mean potassium concentrations (mEq/L) in the circulating blood surrogate solution collected after exposure to a dialyzer were 1.4 ± 0.3, 1.6 ± 0.3, 1.5 ± 0.1, 1.2 ± 0.1 at 0, 15, 30, and 60 minutes, respectively (p = 0.925 for comparison between the baseline and mean of levels from the 15, 30 and 60 minute timepoints and p = 0.365 for comparison across the timepoints of 0, 15, 30, and 60 minutes of sodium thiosulfate infusion). Mean potassium concentrations (mEq/L) in the circulating blood surrogate solution collected before exposure to a dialyzer were statistically not different (p = 0.163 for comparison between the baseline and mean of levels from the 15, 30 and 60 minute timepoints and p = 0.350 for comparison across the timepoints of 0, 15, 30, and 60 minutes of sodium thiosulfate infusion).

**Fig 5:**
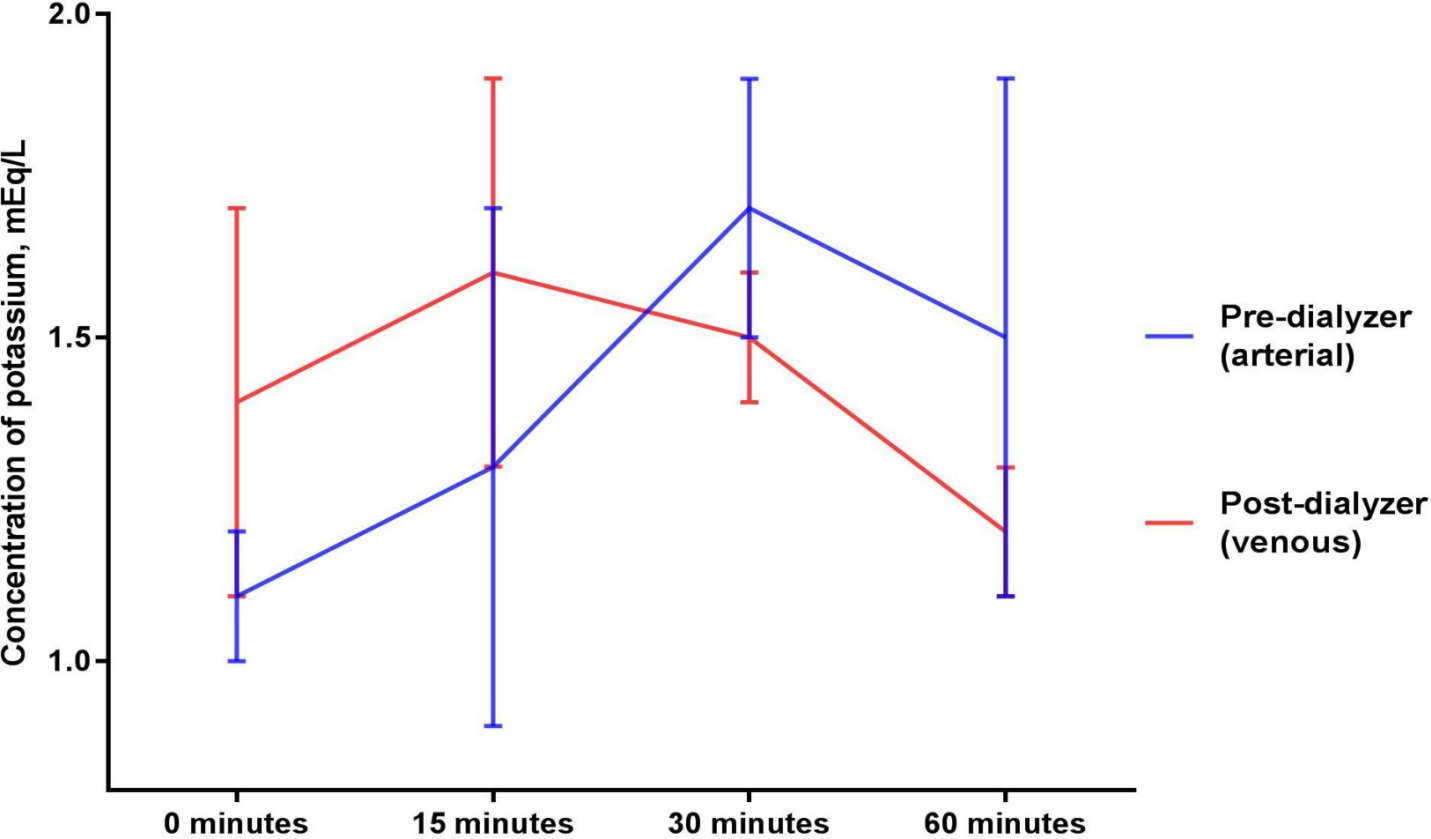
Potassium concentrations in the circulating blood surrogate solution before and after exposure to a dialyzer during ongoing hemodialysis and 60-minute infusion of 25 g Sodium Thiosulfate Injection.

**Fig 6:**
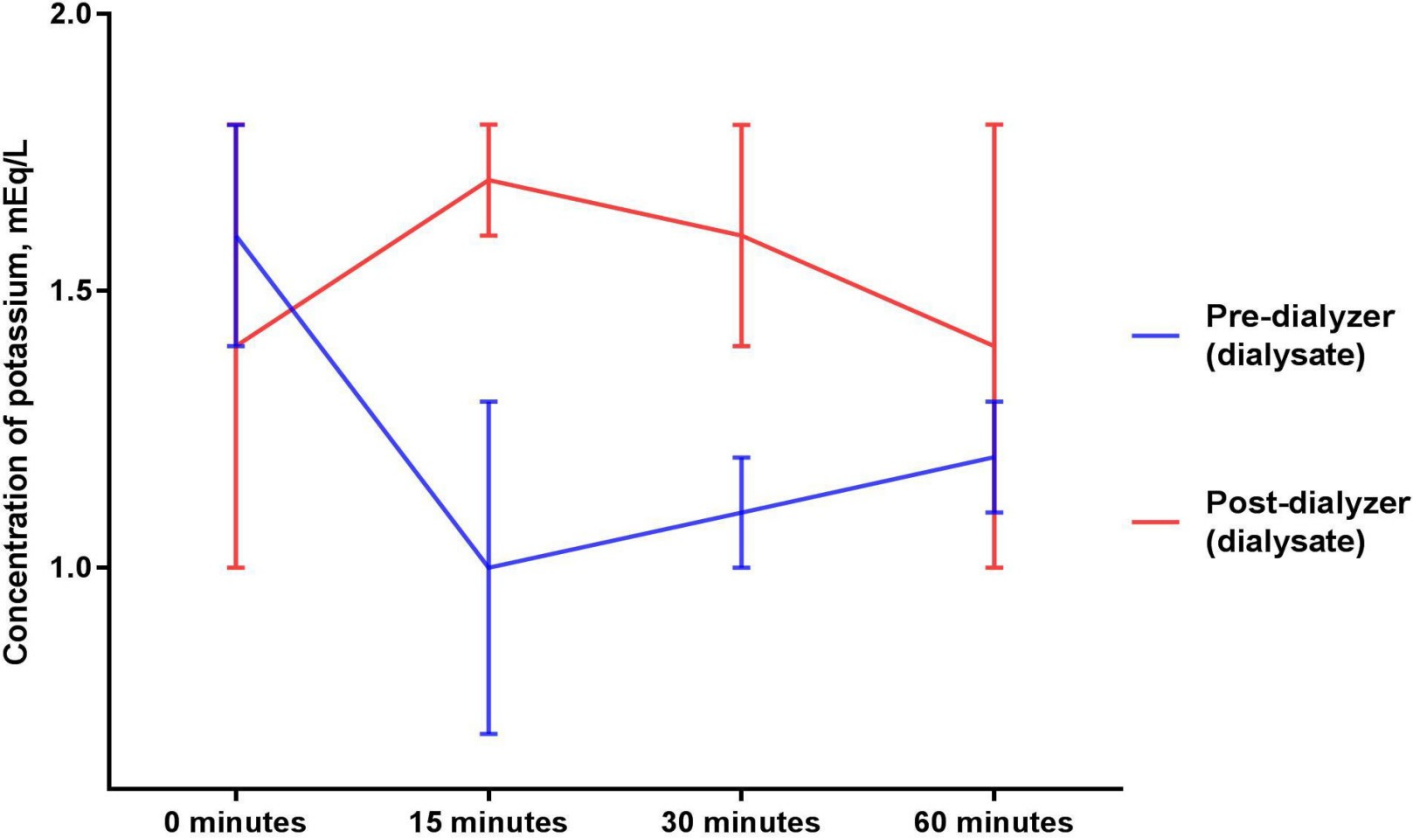
Potassium concentrations in dialysate before and after exposure to a dialyzer during ongoing hemodialysis and 60-minute infusion of 25 g Sodium Thiosulfate Injection.

Potassium concentrations in dialysate increased marginally after exposure to the dialyzer (p = 0.062 for comparison between the baseline and mean of levels from the 15, 30 and 60 minute timepoints and p = 0.199 for comparison across the timepoints of 0, 15, 30, and 60 minutes of sodium thiosulfate infusion).

A Supporting Information file includes the datasets for thiosulfate, sodium, and potassium measurements.

## Discussion

Potassium and sodium are excreted from the body primarily by the kidneys. Subjects with end-stage kidney disease have significantly reduced elimination of potassium and sodium; thereby necessitating chronic renal replacement therapy and careful management of potassium and sodium exposures.

Control of sodium and potassium are crucial functions of dialysis. Our experiment used an *in vitro* hemodialysis model to examine whether hemodialysis can remove from blood the incremental exposures to sodium and potassium that are associated with sodium thiosulfate therapy. This study confirmed our hypothesis that neither potassium nor sodium accumulated in a circulating blood surrogate solution when a dose of sodium thiosulfate was infused in conjunction with hemodialysis and rates of ultrafiltration and sodium thiosulfate infusion were maintained as identical. The concentrations of both ions were increased marginally in spent dialysate indicating that potassium and sodium ions that were introduced by the medication were likely removed from the circulating blood surrogate solution by hemodialysis.

Our model controlled for many of the variables that affect dialytic clearance, such as hemodialyzer type and flow rates. This model has been used to measure the clearance of other medications during hemodialysis and is a predictor of *in vivo* drug clearance and accumulation.[14] Sodium thiosulfate has a substantial dialytic clearance.[15] Our approach of adding sodium thiosulfate solution to the circulating blood surrogate solution using an infusion pump not only simulated a typical clinical scenario of intravenous sodium thiosulfate administration into the venous blood line but also eliminated the possibility of much larger clearances of thiosulfate, sodium and potassium linked with the infusion of sodium thiosulfate into an arterial bloodline. We applied a 5-liter beaker to simulate typical human blood volume. We acknowledge, however, that the infusion of sodium thiosulfate into venous bloodline of dialyzer in humans may result in distribution of sodium thiosulfate into a physiological space larger than typical blood volume of 5 liters. A study that applies full blood is needed to confirm our results. A clinical study that records concentrations of sodium and potassium and changes in volume status of human subjects receiving sodium thiosulfate infusion is being conducted to confirm our findings.^9^ This is particularly relevant as previous retrospective data suggest that there is mild elevation in serum sodium concentration (on average 1–2 mEq/L) in patients receiving sodium thiosulfate infusion therapy for calciphylaxis.[4, 16] Sample collection timepoints, dose of sodium thiosulfate, and other variables that may impact serum sodium concentration in these prior retrospective studies were not standardized and thus, robust clinical inferences could not be drawn and future more rigorous studies are needed. Moreover, the impact of sodium thiosulfate infusion on electrolyte concentrations including bicarbonate needs evaluation and is being pursued in the ongoing phase 3 study.^9^

In conclusion, our *in vitro* study demonstrates that neither potassium nor sodium accumulates in a circulating blood surrogate solution when a dose of sodium thiosulfate is infused in conjunction with hemodialysis.

## Supporting information

S1 Table Sodium Thiosulfate concentrations. Sodium thiosulfate concentrations in the circulating blood surrogate solution.
(PDF)

S2 Table Sodium concentrations. Sodium concentrations in the circulating blood surrogate and dialysate solutions.
(PDF)

S3 Table Potassium concentrations. Potassium concentrations in the circulating blood surrogate and dialysate solutions.
(PDF)
