# Supplementary material for: Impact of hemodialysis on the concentrations of sodium and potassium during infusion of sodium thiosulfate using an In Vitro hemodialysis model
Source: PLoS One. 2019 Nov 13;14(11):e0224767. doi: 10.1371/journal.pone.0224767 (PMC6853332; doi:10.1371/journal.pone.0224767)
Supplement: S1 Table — Sodium thiosulfate concentrations in the circulating blood surrogate solution. (PDF) [file pone.0224767.s001.pdf]

S1 Table. Sodium Thiosulfate Concentrations. Sodium thiosulfate concentrations in the circulating blood surrogate solution.

Designation	Blood Surrogate Solution Sodium Thiosulfate Concentration (µg/mL)
0 Minute Pre-Filter Arterial	0
	0
	0
15 Minute Pre-Filter Arterial	423.2
	413.5
	330.1
30 Minute Pre-Filter Arterial	1198.5
	751.1
	1156.6
1 Hour Pre-Filter Arterial	1184.4
	662
	1089.7
0 Minute Post-Filter Arterial	0
	0
	0
15 Minute Post-Filter Venous	256.7
	37.4
	32.6
30 Minute Post-Filter Venous	186.6
	47
	85.5
1 Hour Post-Filter Venous	43.6
	43.6
	174.4
