# Supplementary material for: Impact of hemodialysis on the concentrations of sodium and potassium during infusion of sodium thiosulfate using an In Vitro hemodialysis model
Source: PLoS One. 2019 Nov 13;14(11):e0224767. doi: 10.1371/journal.pone.0224767 (PMC6853332; doi:10.1371/journal.pone.0224767)
Supplement: S2 Table — Sodium concentrations in the circulating blood surrogate and dialysate solutions. (PDF) [file pone.0224767.s002.pdf]

S2 Table. Sodium Concentrations. Sodium concentrations in the circulating blood surrogate and dialysate solutions.

Designation	Blood Surrogate Solution Sodium Concentration (mEq/L)	Designation	Dialysate Solution Sodium Concentration (mEq/L)
0 Minute Pre-Filter Arterial	81	0 Minute Pre-Filter Dialysate	132.3
	99.8		97.1
	84.7		126.4
15 Minute Pre-Filter Arterial	101.2	15 Minute Pre-Filter Dialysate	59.4
	131.7		87.6
	64.9		110.2
30 Minute Pre-Filter Arterial	133.4	30 Minute Pre-Filter Dialysate	100.1
	111.5		83.6
	117.8		92.6
1 Hour Pre-Filter Arterial	109.7	1 Hour Pre-Filter Dialysate	105.7
	70.2		87.5
	142.6		99.1
0 Minute Post-Filter Venous	113.8	0 Minute Post-Filter Dialysate	76
	85		144.2
	110.9		95.7
15 Minute Post-Filter Venous	119.7	15 Minute Post-Filter Dialysate	140.3
	86		131.5
	136.8		121.8
30 Minute Post-Filter Venous	119.8	30 Minute Post-Filter Dialysate	125.1
	105.9		110
	125.9		126.6
1 Hour Post-Filter Venous	102.4	1 Hour Post-Filter Dialysate	89.8
	85.9		146.4
	92.2		95.4
