# Supplementary material for: Impact of hemodialysis on the concentrations of sodium and potassium during infusion of sodium thiosulfate using an In Vitro hemodialysis model
Source: PLoS One. 2019 Nov 13;14(11):e0224767. doi: 10.1371/journal.pone.0224767 (PMC6853332; doi:10.1371/journal.pone.0224767)
Supplement: S3 Table — Potassium concentrations in the circulating blood surrogate and dialysate solutions. (PDF) [file pone.0224767.s003.pdf]

S3 Table. Potassium Concentrations. Potassium concentrations in the circulating blood surrogate and dialysate solutions.

Designation	Blood Surrogate Solution Potassium Concentration (mEq/L)	Designation	Dialysate Solution Potassium Concentration (mEq/L)
0 Minute Pre-Filter Arterial	1.1	0 Minute Pre-Filter Dialysate	1.8
	1.3		1.2
	1		1.7
15 Minute Pre-Filter Arterial	1.5	15 Minute Pre-Filter Dialysate	0.6
	1.8		1
	0.7		1.4
30 Minute Pre-Filter Arterial	2	30 Minute Pre-Filter Dialysate	1.2
	1.5		1
	1.6		1.1
1 Hour Pre-Filter Arterial	1.6	1 Hour Pre-Filter Dialysate	1.3
	1		1.1
	2		1.2
0 Minute Post-Filter Venous	1.8	0 Minute Post-Filter Dialysate	1
	1		1.9
	1.4		1.2
15 Minute Post-Filter Venous	1.8	15 Minute Post-Filter Dialysate	1.9
	1.1		1.7
	1.8		1.6
30 Minute Post-Filter Venous	1.6	30 Minute Post-Filter Dialysate	1.8
	1.4		1.4
	1.6		1.7
1 Hour Post-Filter Venous	1.3	1 Hour Post-Filter Dialysate	1.1
	1.1		2
	1.1		1.1
